# Primary duodenal malignant melanoma: A case report

**DOI:** 10.22088/cjim.9.3.312

**Published:** 2018

**Authors:** Kazem Anvari, Masoumeh Gharib, Amir Hossein Jafarian, Amin Saburi, Seyed Alireza Javadinia

**Affiliations:** 1 Cancer Research Center, Faculty of Medicine, Mashhad University of Medical Sciences, Mashhad, Iran; 2 Department of Pathology, Faculty of Medicine, Mashhad University of Medical Sciences, Mashhad, Iran; 3 Birjand Atherosclerosis and Coronary Artery Research Center, Birjand University of Medical Sciences, Birjand, Iran; 4 Student Research Committee, Faculty of Medicine, Mashhad University of Medical Sciences, Mashhad, Iran.

**Keywords:** Melanoma, Duodenum, Gastrointestinal tract

## Abstract

**Background:**

Melanoma is a neoplasm derived commonly from melanocytic cells of skin. Although coetaneous presentation of malignant melanoma is easily recognizable, the presentation of melanoma in other organs is so confusing. In particular, when it metastasizes to other organs, many bizarre figures and unusual organs may be involved. In this report, we present a case of primary duodenal malignant melanoma.

**Case Presentation:**

A 68-year-old man presented with a history of iron deficiency anemia. The upper gastrointestinal endoscopy showed a prominent papilla of duodenum along with an ulcerative lesion adjacent to second part of duodenum. Histopathologic evaluation showed a high-grade malignant neoplasm involving the bowel wall which was labeled for S100 protein and markers of melanocytic differentiation; Melan-A indicating the definitive diagnosis of malignant melanoma of the second portion of duodenal mucosa.

**Conclusions:**

In patients with a history of iron deficiency anemia, any GI symptom should be evaluated carefully. However, the diagnosis of primary GI melanomas in patients without any history of melanoma is possible. Full medical investigations are recommended in these patients with primary mucosal lesions.

Malignant melanoma is commonly diagnosed on skin lesions    (1). This cancer is one of the most aggressive and life-threatening skin cancers. Malignant melanoma originate from the melanocytes and has a very high tendency to spread to other parts of the body. However, involvement of extra-cutaneous sites; such as gastrointestinal mucosa which may contain pigmented cells/melanocytes, are well documented in the literature (-). Unlike cutaneous melanoma, which is one of the most common malignancies with a significant burden on society and public health globally (5), primary mucosal melanomas are rare(4). Mucosal melanoma originating in duodenum with gallbladder metastasis is a very rare condition, which presents non-specific signs and symptoms like other pathological conditions of this region. Here, we present a-68-year old man who presented with fatigue and iron deficiency anemia. After upper gastrointestinal endoscopy and biopsy, pathological and immunohistochemical studies confirmed the diagnosis of duodenal malignant melanoma. 

## Case Presentation

A 68-year-old man was referred to our oncology clinic with the pathologic diagnosis of duodenal malignant melanoma. This patient had a history of controlled diabetes mellitus, controlled hypertension and myocardial infraction before admission. He had suffered from fatigue, weakness, lethargy and weight lost (more than 10%) since one month prior to admission. Physical examination revealed no additional clinical data. 

A complete blood count (CBC) test showed an iron deficiency anemia. Thyroid function tests were normal. Moreover, he underwent an upper gastrointestinal endoscopy (GIE) and a colonoscopy that revealed some erosion around pylorus of stomach and a prominent papilla of duodenum along with an ulcerative lesion adjacent to D2. Several biopsies were taken from the antrum and D2 lesions.

**Figure 1:**
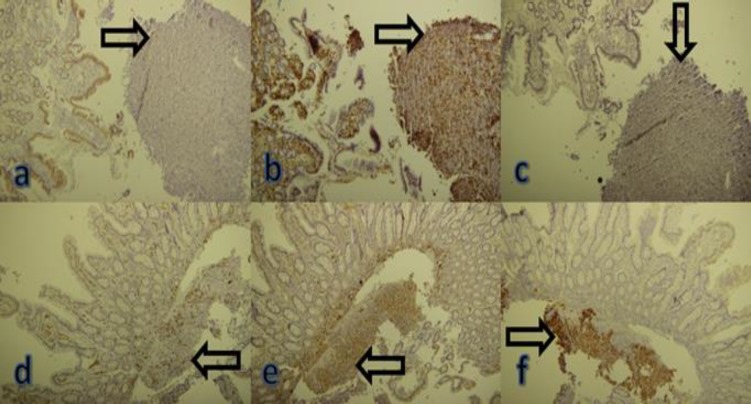
IHC; a-d: CK, LCA, CD117, and CD34 were negative. e and f: S100 protein and Melan-A were positive. The tumor cells labeled for S100 protein and markers of melanocytic differentiation; Melan-A. As staining for CK, LCA, CD117, and CD34 were negative, the diagnosis of carcinoma, lymphoma and gastrointestinal stromal tumor were ruled out (red arrows showing the tumoral cells)

**Figure 2:**
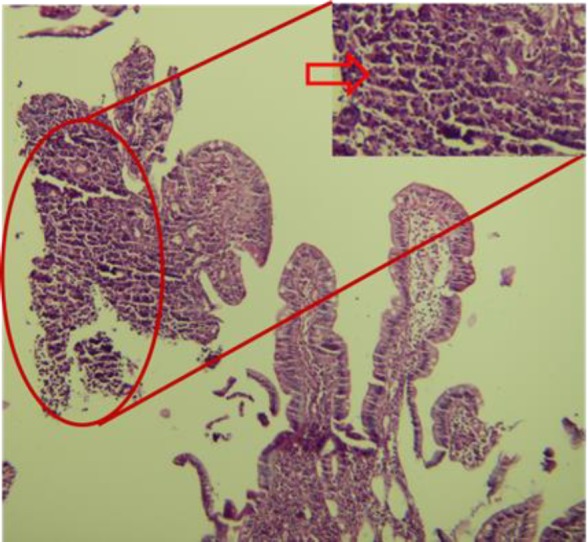
Histopathologic evaluation with H&E staining; a high-grade malignant neoplasm involving the bowel wall. Tumor was composed of sheets of loosely cohesive pleomorphic cells with prominent nucleoli and eosinophilic cytoplasm (red oval shape represents region occupied by tumor cells where the red arrow showing the tumoral cell).

Histopathologic evaluation showed a high-grade malignant neoplasm involving the bowel wall. Tumor was composed of sheets of loosely cohesive pleomorphic cells with prominent nucleoli and eosinophilic cytoplasm. There was no visible melanin pigment in tumoral cells. Necrosis was also noted. The tumor cells labeled for S100 protein and markers of melanocytic differentiation; Melan-A. As staining for CK, LCA, CD117, and CD34 were negative, the diagnoses of carcinoma, lymphoma and gastrointestinal stromal tumor were ruled out. Morphologic and immunohistochemical findings were consistent with malignant melanoma (figures 1 and 2).

**Figure 3:**
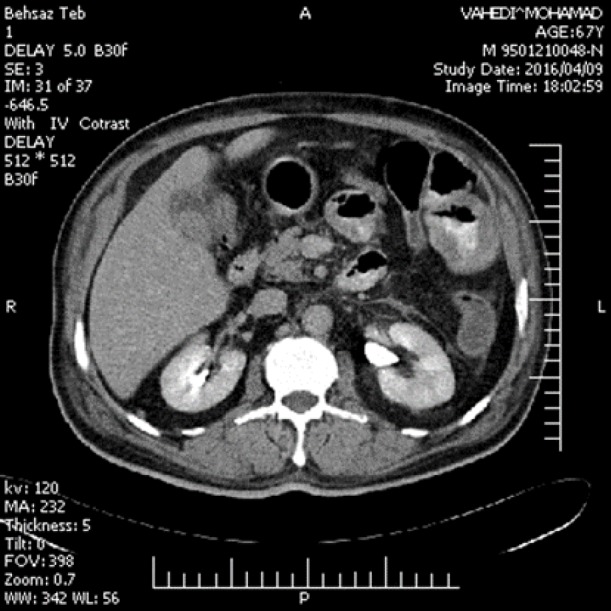
Abdominopelvic computed tomography (CT) scan with intravenous contrast; Abdominopelvic computed tomography (CT) scan with intravenous contrast revealed multiple abnormalities. CT scan exhibited an ovaloid mass in the gallbladder with washout in delayed phase that was suggestive of a tumoral lesion. There were two small nodules in the right adrenal and a heterogeneous hypodense mass (diameter: 3.2 cm) in the left adrenal. In the proximal (and to a lesser extent in distal) loops of the small intestine, a heterogeneous increase in thickness was also observed. Moreover, there were several mesenteric lymphadenopathies along the superior mesenteric artery (figure 3)

Subsequently, after confirming the diagnosis of malignant melanoma of duodenal mucosa, the patient underwent secondary full medical evaluation including detailed inspection of the eyes, skin and mucosal surface. These examinations did not show any clinically significant lesion. Moreover, he underwent anoscopy for any visible lesion that was negative. Abdominopelvic computed tomography (CT) scan with intravenous contrast revealed multiple abnormalities. CT scan exhibited an ovaloid mass in the gallbladder with washout in delayed phase that was suggestive of a tumoral lesion. There were two small nodules in the right adrenal and a heterogeneous hypodense mass (diameter: 3.2 cm) in the left adrenal. In the proximal (and to a lesser extent in distal) loops of the small intestine, a heterogeneous increase in thickness was also observed. Moreover, there were several mesenteric lymphadenopathies along the superior mesenteric artery (figure 3).

Considering the pathologic diagnosis of the duodenal lesion and its metastatic nature, we commenced treatment of patient with oral temozolomide (every 28 days) and scheduled further diagnostic interventions including CT guided fine needle aspiration biopsy of the abdominal mass and re-upper GIE, unfortunately he expired due to active disease.

## Discussion

Malignant melanoma is a rare skin cancer causing the highest rate of mortality among them. There is a geographical variation for melanoma incidence. However, its incidence increased during last decades (6). In contrast to these facts about cutaneous types, the prevalence of mucosal melanomas remains steady and they account for up to 2% of all melanomas mostly in esophagus and anus  (7, 8).

Melanoma originates from pigment-producing cells –melanocytes- which are mainly located at the skin, eyes, and meninges. Furthermore, melanocytes exist in mucosal surfaces except in the small intestine mucosa (4). Cutaneous melanoma usually spread to lung, liver, brain and less gastrointestinal (GI) tract (that it mostly metastasie to small intestine). Gallbladder metastasis of melanoma is an extremely rare medical condition which was described mostly as case reports (9). Although, there are some reports of primary melanomas of these organs, the main pathophysiology of these primary mucosal lesions is unclear. Nonetheless, some investigators such as Mishimaet.Al. (10) and Amar et.Al. (11) suggest schwannian neuroblast cells and melanoblastic cells of the neural crest, the cell of origin. 

Mucosal melanoma originating in duodenum presents a broad spectrum of clinical features such as abdominal pain, intestinal obstruction, constipation, blood vomiting, melena, anemia, fatigue, weight loss, and presence of a palpable abdominal mass (12, 13). These clinical presentations mostly resemble ulcerative lesions of gastrointestinal tract. Regarding to its pathohistology, there is no expectation to present with GI bleeding; there are some reports about the first presentation of duodenal melanoma with this rare presentation (14). Moreover, according to the location of GI tract, it can be present with complicated diagnosis such as painless jaundice (15). Nevertheless, other differential diagnosis include gastritis, esophageal varices, Meckel's diverticulum, and Mallory-Weiss syndrome. These various and different symptoms make the differentiation of gastrointestinal metastatic or primary mucosal melanomas very difficult (16). Upper GIE is the procedure of choice in the detection of mucosal melanomas of duodenum, as it is for other pathologies of upper GI tract. Ettahri et Al. (9) described endoscopic findings of malignant melanoma of GI tract as necrotic/melanotic lesions, sub-mucosal masses, and ulcerated mucosal melanoma. Like the presented patient, the upper GIE showed an ulcerative lesion in D2. On the other hand, primary malignant melanoma of the duodenum can be a mimicker of lymphoma or carcinoma when it is without visible melanin pigment (17). In patients with duodenal melanoma, full medical investigations include detailed examination of eyes, skin and mucosal surfaces, endoscopy, abdominopelvic CT scan with intravenous contrast, and positron emission tomography–computed tomography (PET/CT) scan are imperative. However, since cutaneous melanoma usually tends to regress (9), these diagnostic procedures may fail in detecting the primary lesion. moreover, these tumors may mimic other neoplasms like masquerading as gastrointestinal stromal tumor (18). As this medical condition is rare, its pathologic diagnosis needs immunohistochemical confirmation by HMB45, Melan-A and S-100 protein (4, 13). A complete immunohistochemical panel could result in the highest possible sensitivity and specificity.

In conclusion in patients with a history of melanoma, any GI symptom should be evaluated carefully. Yet, the diagnosis of primary GI melanomas in patients without any history of melanoma is possible. Full medical investigations are recommended in these patients with primary mucosal lesions. 

**Compliance with Ethical Standards**

Written informed consent was obtained from the patient for the ublication of this case report and any accompanying images. A copy of the written consent is available for review by the Editor-in-Chief of this journal. All procedures performed in studies involving human participants were in accordance with the ethical standards of the institutional and/or national research committee and with the 1964 Helsinki declaration and its later amendments or comparable ethical standards. 

## References

[1] Salehiniya H, Ghobadi-Dashdebi S, Rafiemanesh H, Mohammadian-Hafshejani A, Enayatrad M (2016). Time trend analysis of cancer‏ incidence in Caspian Sea, 2004–2009: a population-based cancer registries study (northern Iran). Caspian J Intern Med.

[2] Suganuma T, Fujisaki J, Hirasawa T (2013). Primary amelanotic malignant melanoma of the small intestine diagnosed by esophagogastroduodenoscopy before surgical resection. Clin J Gastroenterol.

[3] Li H, Fan Q, Wang Z (2012). Primary malignant melanoma of the duodenum without visible melanin pigment: a mimicker of lymphoma or carcinoma. Diagn Pathol.

[4] Mihajlovic M, Vlajkovic S, Jovanovic P, Stefanovic V (2012). Primary mucosal melanomas: a comprehensive review. Int J Clin Exp Pathol.

[5] Nikolaou V, Stratigos A (2014). Emerging trends in the epidemiology of melanoma. Br J Dermatol.

[6] Forsea AM, Del Marmol V, De Vries E, Bailey E, Geller A (2012). Melanoma incidence and mortality in Europe: new estimates, persistent disparities. Br J Dermatol.

[7] McLaughlin CC, Wu XC, Jemal A (2005). Incidence of noncutaneous melanomas in the US. Cancer.

[8] Chang AE, Karnell LH, Menck HR (1998). The National Cancer Data Base report on cutaneous and noncutaneous melanoma. Cancer.

[9] Ettahri H, Elomrani F, Elkabous M (2015). Duodenal and gallbladder metastasis of regressive melanoma: a case report and review of the literature. J Gastrointest Oncol.

[10] Mishima Y (1967). Melanocytic and nevocytic malignant melanomas cellular and subcellular differentiation. Cancer.

[11] Amar A, Jougon J, Edouard A, Laban P, Marry J, Hillion G (1991). Primary malignant melanoma of the small intestine. Gastroenterol Clin Biol.

[12] Patti R, Cacciatori M, Guercio G, Territo V, Di Vita G (2012). Intestinal melanoma: A broad spectrum of clinical presentation. Int J Surg Case Rep.

[13] Lens M, Bataille V, Krivokapic Z (2009). Melanoma of the small intestine. Lancet Oncol.

[14] Çoban Ş, Ekiz F, Başar Ö (2014). A rare cause of upper gastrointestinal bleeding in an elderly patient: primary duodenal malignant melanoma. J Gastrointest Cancer.

[15] Bendic A, Durdov MG, Stipic R, Karaman I (2013). Melanoma in the ampulla of Vater. Hepatobiliary Pancreatic Dis Int.

[16] Reintgen D, Thompson W, Garbutt J, Seigler H (1984). Radiologic, endoscopic, and surgical considerations of melanoma metastatic to the gastrointestinal tract. Surgery.

[17] Li H, Fan Q, Wang Z (2012). Primary malignant melanoma of the duodenum without visible melanin pigment: a mimicker of lymphoma or carcinoma. Diagn Pathol.

[18] Gabali AM, Priebe P, Ganesan S (2008). Primary melanoma of small intestine masquerading as gastrointestinal stromal tumor: a case report and literature review. Am Surg.

